# A NET Outcome

**DOI:** 10.3389/fimmu.2012.00365

**Published:** 2012-12-05

**Authors:** Thea Lu, Scott D. Kobayashi, Mark T. Quinn, Frank R. DeLeo

**Affiliations:** ^1^Laboratory of Human Bacterial Pathogenesis, Rocky Mountain Laboratories, National Institute of Allergy and Infectious Diseases, National Institutes of HealthHamilton, MT, USA; ^2^Department of Immunology and Infectious Diseases, Montana State UniversityBozeman, MT, USA

**Keywords:** neutrophil, apoptosis, necrosis, phagocytosis, inflammation

## Abstract

Neutrophils constitute a critical part of innate immunity and are well known for their ability to phagocytose and kill invading microorganisms. The microbicidal processes employed by neutrophils are highly effective at killing most ingested bacteria and fungi. However, an alternative non-phagocytic antimicrobial mechanism of neutrophils has been proposed whereby microorganisms are eliminated by neutrophil extracellular traps (NETs). NETs are comprised of DNA, histones, and antimicrobial proteins extruded by neutrophils during NETosis, a cell death pathway reported to be distinct from apoptosis, phagocytosis-induced cell death, and necrosis. Although multiple laboratories have reported NETs using various stimuli *in vitro*, the molecular mechanisms involved in this process have yet to be definitively elucidated, and many questions regarding the formation and putative role or function of NETs in innate host defense remain unanswered. It is with these questions in mind that we provide some reflection and perspective on NETs and NETosis.

## Neutrophil Turnover and Homeostasis

Neutrophils are short-lived granulocytes that mature in bone marrow for several days (Bainton et al., 1971; Weissman et al., 2001). During maturation, these cells acquire key functional attributes, including the ability to phagocytose and kill microorganisms (Bainton et al., 1971; Glasser and Fiederlein, 1987; Weissman et al., 2001; Rosenbauer and Tenen, 2007; Pillay et al., 2010). After maturation, neutrophils are released into the bloodstream and circulate and/or marginate for 10–24 h before migrating into tissues, where they may function for an additional 1–2 days before they undergo apoptosis and are cleared by macrophages or dendritic cells (Cartwright et al., 1964; Fliedner et al., 1964; Bainton et al., 1971; Savill et al., 1989; Voll et al., 1997; Fadok et al., 1998; Huynh et al., 2002; Martin et al., 2003; Rigby and DeLeo, 2012). In addition, neutrophils in the total blood granulocyte pool (circulating and marginating) can be removed by the liver, spleen, and bone marrow, although the precise mechanism for this turnover process remains incompletely determined (reviewed by Summers et al., 2010). The neutrophil lifespan is highly regulated, as it is critical to remove spent/effete neutrophils as a means to prevent accidental release of cytotoxic molecules and associated host tissue damage (Edwards et al., 2003; Duffin et al., 2010; Bratton and Henson, 2011; Milot and Filep, 2011). Neutrophil turnover in an adult human is typically on the order of 10^11^ cells per day (Athens et al., 1961; Dancey et al., 1976; Rankin, 2010). While the hematopoietic system is able to regulate steady-state levels of circulating neutrophils, it can also be switched to an emergency granulopoiesis response to accommodate the increased demand for neutrophils during infection (Hirai et al., 2006; Panopoulos and Watowich, 2008).

The neutrophil lifespan is regulated by a balance of pro- and anti-apoptotic factors present in the environment. Cytokines and other factors such as interleukin (IL)-1β, IL-2, IL-4, IL-15, interferon-γ, granulocyte colony-stimulating factor (G-CSF), granulocyte-macrophage colony-stimulating factor (GM-CSF), and lipopolysaccharide (LPS) can prolong and/or enhance neutrophil function and delay apoptosis for several days (Colotta et al., 1992; Duffin et al., 2010). Although enhancing neutrophil function and survival presumably favors elimination of invading microbes, the persistence of these cytotoxic host cells increases the potential for prolonged inflammation and host tissue damage. Therefore, it is not surprising that neutrophil turnover is a highly regulated process.

Molecular control of neutrophil turnover or apoptosis is mediated by several mechanisms, including extrinsic pathways induced by extracellular signals and intrinsic pathways induced by intracellular signals. These signals include those triggered by death receptors, which bind ligands that activate caspases to promote apoptosis, mitochondrial release of cytochrome *c*, and processes mediated by the BCL-2 protein family (Edwards et al., 2003; Duffin et al., 2010). Spontaneous or constitutive apoptosis in neutrophils is an example of intrinsic apoptosis. Apoptosis elicited by FAS, tumor necrosis factor (TNF)-α, or TNF-related apoptosis inducing ligand (TRAIL), caused by the binding of these extracellular ligands to the cognate receptor anchored on the cell surface, is an example of extrinsic pathway apoptosis (Kennedy and DeLeo, 2009; Duffin et al., 2010). Phagocytosis may also lead to neutrophil apoptosis (Watson et al., 1996; Kobayashi et al., 2002; Zhang et al., 2003; Kennedy and DeLeo, 2009). Neutrophil phagocytosis-induced apoptosis or phagocytosis-induced cell death (PICD) promotes the resolution of infection by disposing spent or effete neutrophils containing dead or partially digested microbes in a non-inflammatory manner (Kennedy and DeLeo, 2009). This process is described below in the context of the resolution of inflammation.

## Neutrophils and the Inflammatory Response

The importance of neutrophils in the immune response is underscored by human diseases caused by defects in neutrophil function, which result in increased risk of infection from bacteria and fungi (Nauseef and Clark, 2010). For example, neutropenia, which can be medically induced by cytotoxic drugs or cancer therapy, is associated with significant morbidity (Bodey et al., 1966; Dale et al., 1979; Frøland, 1984; Tobias and Schleien, 1991). In addition, the inflammatory response, which from a cellular perspective is largely comprised of neutrophils, is critical for defense against invading microorganisms. On the other hand, timely resolution of the inflammatory response is an important process that returns the host immune system to pre-infection homeostasis. Historically, neutrophils were considered to have a passive part in inflammation resolution; however, this view has changed over time, and it is now known that neutrophils actively help to resolve inflammation by blocking and scavenging chemokines and cytokines (Ariel et al., 2006), and also produce pro-resolving lipid mediators (Ariel et al., 2006; Serhan et al., 2008). Thus, given that neutrophils contain and produce a vast array of cytotoxic molecules and contribute to the regulation of inflammation, it should not be unexpected that these cells are involved in – or are the primary cause of – a variety of inflammatory disorders. For instance, in chronic obstructive pulmonary disease, the aminopeptidase activity of leukotriene A4 hydrolase (LTA4H) is inhibited, causing accumulation of proline-glycine-proline, which in turn, promotes neutrophil recruitment and chronic lung inflammation (Weathington et al., 2006). In mouse models, recruitment of neutrophils has been shown to be involved in arthritis (Chou et al., 2010) and multiple sclerosis (Carlson et al., 2008; Liu et al., 2010). More notably, recent studies have demonstrated that neutrophils and neutrophil responses (rather than bacterial pathogens *per se*) are the cause of severe pneumonia and tissue destruction in animal models of bacterial respiratory tract infection (Bartlett et al., 2008; Diep et al., 2010). Thus, it is clear that unchecked neutrophil activation and neutrophil lysis are phenomena that can have a significant negative impact on health of the host.

## Response to Infection

Neutrophils are recruited rapidly to the site of infection in response to chemotactic stimuli released by the host and/or invading microorganism. Inasmuch as neutrophils are the most abundant leukocyte in humans, there can be a tremendous influx of neutrophils to the site of infection. At such sites, neutrophils bind and ingest microorganisms through a process known as phagocytosis (reviewed in Rigby and DeLeo, 2012). Ingested microbes are typically destroyed by the combined effects of NADPH oxidase-derived reactive oxygen species (ROS) and cytotoxic molecules delivered from cytoplasmic granules into the phagosome. Neutrophil granules contain numerous antimicrobial peptides (AMPs) and proteins, and matrix protein-degrading proteases, including alpha-defensins, cathelicidins, azurocidin, cathepsins, lactoferrin, lysozyme, proteinase-3, gelatinase, collagenase, and elastase (Faurschou and Borregaard, 2003; Nauseef and Clark, 2010; Rigby and DeLeo, 2012). Of note, these cytotoxic agents are normally targeted into the formed phagosome, thereby limiting inadvertent extracellular release and potential damage to host tissues (Nauseef and Clark, 2010).

**Figure 1:**
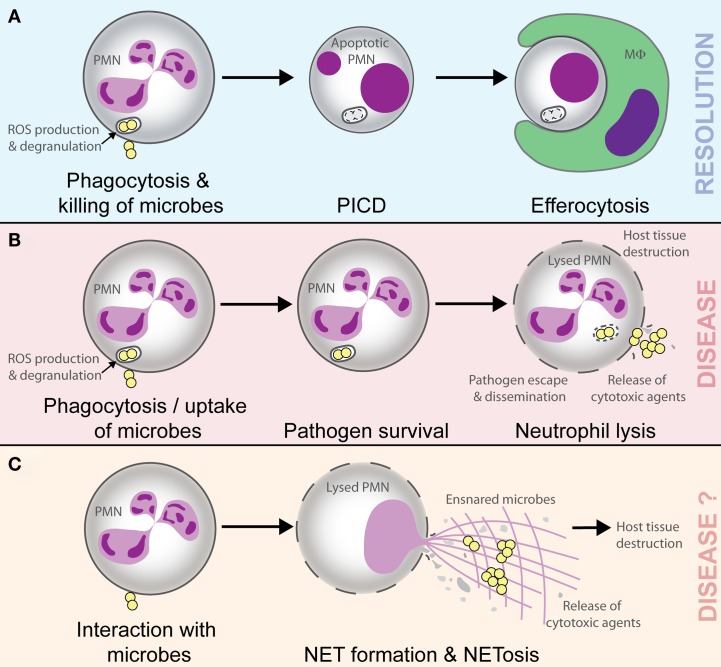
**Possible outcomes of the interaction of microbes with neutrophils**. Phagocytosis and killing of microorganisms by neutrophils (polymorphonuclear leukocyte, PMN) triggers host cell apoptosis and ultimate removal by macrophages (MΦ) or dendritic cells. This process promotes resolution of the inflammatory response **(A)**. Pathogenic microbes such as *Staphylococcus aureus* can cause lysis of PMN after phagocytosis, thereby facilitating escape/dissemination of the invading pathogen and release of cytotoxic molecules that cause host tissue damage and disease **(B)**. NETs ensnare and may kill microbes, but there is accompanying lysis of neutrophils and release of cytotoxic molecules that are known to cause host tissue damage and promote inflammatory disease. In this regard, the outcome of NETosis and the formation of NETs should be similar to that in **(B**; i.e., disease; **C)**.

It is well documented that neutrophil PICD occurs following ingestion of numerous microorganisms *in vitro* (Watson et al., 1996; Colamussi et al., 1999; Engelich et al., 2001; Kobayashi et al., 2003a,b, 2012; Kennedy and DeLeo, 2009), and *in vivo* this phenomenon likely promotes clearance of effete neutrophils containing dead or dying microbes (Figure 1; Kobayashi et al., 2012). Importantly, this process would prevent local host tissue damage that can occur if these spent host cells are not removed and undergo lysis, and thus ultimately promotes the resolution of inflammation (Whyte et al., 1993; Savill, 1997; Kobayashi et al., 2002, 2003a, 2012; Kim et al., 2004; Iyoda et al., 2005; Ariel et al., 2006; Kobayashi and DeLeo, 2009; Rigby and DeLeo, 2012). Such a process is considered normal for neutrophils during infection and healthy for the host. On the other hand, pathogenic microorganisms circumvent killing by neutrophils, and in doing so ultimately alter the normal process of neutrophil turnover during infection, by either delaying apoptosis or causing neutrophil lysis (Kobayashi et al., 2003a, 2010; DeLeo, 2004; Voyich et al., 2005). The resulting neutrophil lysis releases tissue-damaging molecules, not only allowing pathogen survival but also exacerbating the inflammatory response (Figure 1). This process can lead to disease and can be considered unhealthy for the host. As one example, some strains of *Staphylococcus aureus* are known to cause lysis of human neutrophils after phagocytosis (Rogers and Tompsett, 1952; Voyich et al., 2005, 2006; Kobayashi et al., 2010). Indeed, the possibility that *S. aureus* survive after phagocytosis and ultimately disseminate to cause disease (which can be explained at least in part by neutrophil lysis after trafficking) has been reviewed recently (Thwaites and Gant, 2011). These authors describe neutrophils as “Trojan horses” for the dissemination or metastasis of *S. aureus* (Thwaites and Gant, 2011). In accordance with the observations *in vitro*, *S. aureus* is an abundant cause of pyogenic infections in humans. Therefore, the ability of *S. aureus* to cause neutrophil lysis is likely a component of virulence.

## Neutrophil Extracellular Traps and NETosis

Until fairly recently, phagocyte biologists were content with a model of neutrophil function in which these phagocytes bind, ingest, and subsequently kill microorganisms. The idea that neutrophils would extrude DNA in a cytolytic process that captures microorganisms was unheard of – until Brinkmann et al. (2004) reported the formation of structures known as neutrophil extracellular traps (NETs). These unique structures, which are discussed in detail in this issue of *Frontiers in Immunology*, are composed of DNA, histones, and antimicrobial proteins, and can ensnare pathogens. Since the report by Brinkmann et al. (2004), extracellular traps have been shown to be produced *in vitro* by a number of different cell types, including neutrophils, mast cells, eosinophils, and endothelial cells (Palić et al., 2007; von Köckritz-Blickwede et al., 2008; Yousefi et al., 2008; Chuammitri et al., 2009; Katzenback and Belosevic, 2009; Aulik et al., 2010; Gupta et al., 2010; Wardini et al., 2010; Webster et al., 2010; Lin et al., 2011; Scapinello et al., 2011). Moreover, recent studies have investigated possible mechanisms for the induction of NETs. For example, it has been reported that formation of NETs requires activation of the Raf-MEK-ERK pathway through protein kinase C (Hakkim et al., 2011) and histone citrullination (Neeli et al., 2008; Li et al., 2010; Hemmers et al., 2011; Leshner et al., 2012). These findings suggest that NETosis and formation of NETs involves specific signal transduction events. Thus, it is tempting to advocate the importance of these structures in host defense due to the apparent simplicity and elegance of the phenomenon by which they occur. However, many questions remain about the role of NETs in host defense and the molecular mechanisms underlying their formation are incompletely characterized. In addition, the evidence for formation of NETs *in vivo* is not very compelling, and whether the formation of NETs is of benefit to the host remains an open question. Indeed, it was suggested early on that NETs form only under extreme circumstances and can injure host tissues (Clark et al., 2007).

While use of NETs appears as an alternative mechanism for pathogen control and elimination, production of NETs and NETosis (as a cytolytic process) seems at variance with the highly regulated control of neutrophil turnover (including PICD) and homeostasis, as discussed above. That is, utilization of NETs for host defense contrasts with the considerable effort made by the host to prevent inadvertent neutrophil lysis, release of cytotoxic agents, and post-lysis sequelae, such as inflammatory disorders. Notably, NETs have been implicated in a number of pathologic processes consistent with inflammatory disorders involving lysed neutrophils and cytotoxic molecules from neutrophils. For example, NETs can cause collateral damage in the form of endothelial and tissue damage (Clark et al., 2007; Ma and Kubes, 2008; Marin-Esteban et al., 2012) and may be partially responsible for sputum viscosity and tissue damage in cystic fibrosis patients (Papayannopoulos et al., 2011). NETs have been implicated in systemic lupus erythematosus and systemic vasculitis (Hakkim et al., 2010; Amulic and Hayes, 2011; Bosch, 2011; Garcia-Romo et al., 2011; Lande et al., 2011; Villanueva et al., 2011; Knight and Kaplan, 2012; Liu et al., 2012), gout, asthma, keratinocyte damage, and lupus nephritis (Mitroulis et al., 2011; Marin-Esteban et al., 2012), and may also be involved in the hyper reaction of the immune system by triggering physiological signals and causing pre-eclampsia (Gupta et al., 2005, 2006; Brinkmann and Zychlinsky, 2007). NETs are present in transfusion-related acute lung injury (Thomas et al., 2012), atherosclerotic carotid arteries (Döring, 2012), are toxic to vasculature (Clark et al., 2007; Gupta et al., 2010; Villanueva et al., 2011; Saffarzadeh et al., 2012), and facilitate thrombosis where they could provide a scaffold for red blood cell adhesion (Fuchs et al., 2010, 2012; Van Den Berg and Reitsma, 2011; Brill et al., 2012). NETs may also contribute to cancer-associated thrombosis, since neutrophils from mice with experimentally induced cancers are more likely to form NETs than those from control mice (Demers et al., 2012). It is also of note that extracellular histones, a signature component of NETs, contribute to host death during sepsis (Xu et al., 2009). Whether the structures reported as NETs in these aforementioned inflammatory syndromes are distinct from the remains of necrotic neutrophils is unclear, but in any case the process or phenomenon is associated with a negative outcome for the host – similar to the prediction in the model described in Figure 1.

Given the association of NETs and NETosis with inflammatory disorders, and coupled with a highly regulated neutrophil turnover process, the frequency with which formation of NETs occurs should be fairly low. Indeed, even under optimal NET-inducing conditions *in vitro*, only one-third of activated neutrophils, and perhaps as few as 10%, make NETs (Brinkmann and Zychlinsky, 2007; Fuchs et al., 2007; Munafo et al., 2009). The kinetics of NETosis vary depending on type and concentration of stimulus, isolation procedure of neutrophils, and the sensitivity of the detection method (Fuchs et al., 2012). Despite the fact that NET formation is stimulated by pathogens, it is still not clear whether NETosis that occurs during host-pathogen interactions is a programmed mechanism, a hijacking of host pathways by pathogen-produced factors, or simply an incidental component of neutrophil lysis. For instance, *S. aureus* is well known to cause lysis of neutrophils *in vitro* and *in vivo*, but the pathogen has also been reported to induce NETs (Brinkmann et al., 2004; Jann et al., 2009; Yipp et al., 2012). It is also not clear whether the release of NETs always leads to cell death (and the possibility of host tissue damage) or if it is an extrusion of DNA by intact cells (Yousefi et al., 2009; Remijsen et al., 2011; Guimarães-Costa et al., 2012; Yipp et al., 2012). Although it is difficult to understand how neutrophils can remain intact and viable after release of nuclear DNA, the question of whether NET formation always causes cytolysis or can occur with intact cells is important and must be resolved by the field.

One could hypothesize that the formation of NETs represents a directed host defense mechanism. If the process is host-directed, does this suggest there is an advantage to the use of NETs for removal of microbes versus traditional phagocytosis-based uptake and subsequent killing of microbial invaders? The antimicrobial activities of NETs have been ascribed to the histones, AMPs, and other cytoplasmic components associated with extracellular DNA. However, it is important to note that NETs provide a low concentration of AMPs compared to that present in the phagosome, and NETs lack the ability to produce microbicidal ROS. Published studies to date suggest that the formation of NETs does not lead to the universal killing of all microorganisms, although NETs can reduce the burden of selected of microorganisms *in vitro* (Table 1). This finding is perhaps not surprising, since solubilized azurophilic granule components isolated from disrupted neutrophils have varied capacity to kill different bacterial species (Bertram et al., 1986; Joiner et al., 1989; Levy et al., 1999; Palazzolo-Ballance et al., 2008; Nordenfelt et al., 2009). Moreover, several microorganisms are known to circumvent killing by NETs using a variety of strategies, including altering bacterial surface affinity to NETs (Wartha et al., 2007; Carlin et al., 2009; Juneau et al., 2011) and secreting NET-degrading DNases (Beiter et al., 2006; Buchanan et al., 2006; Midon et al., 2011; Palmer et al., 2011). As an alternative hypothesis, the formation of NETs (especially if it requires lysis of neutrophils) could be considered an incidental event rather than something intended by the host innate immune system. An incidental process seems more consistent with our understanding of the regulation of neutrophil turnover and homeostasis.

**Table 1:** **Microbial susceptibility to NETs**.

Species	Susceptibility	Reference
**VIRUSES**		
Feline leukemia virus	Modulates NET formation	Wardini et al. (2010)
Human immunodeficiency virus (HIV)-1	Infectivity reduced	Saitoh et al. (2012)
Influenza A H1N1	Modulates NET formation	Narasaraju et al. (2011)
**BACTERIA**		
*Actinobacillus suis*	Reduction in bacterial numbers	Scapinello et al. (2011)
*Aeromonas hydrophila*	Survives	Brogden et al. (2012)
*Bacillus anthracis*	Only unencapsulated strains killed	Papayannopoulos and Zychlinsky (2009); Szarowicz and Friedlander (2011)
*Burkholderia pseudomallei*	Reduction in bacterial numbers	Riyapa et al. (2012)
*Escherichia coli*	Reduction in bacterial numbers	Grinberg et al. (2008); Marin-Esteban et al. (2012)
Group A streptococcus	Survives	Buchanan et al. (2006); Lauth et al. (2009)
Group B streptococcus	Survives	Carlin et al. (2009)
*Haemophilus influenzae*	Survives	Juneau et al. (2011)
*Listeria monocytogenes*	Reduction in bacterial numbers	Ramos-Kichik et al. (2009)
*Mannheimia haemolytica*	Reduction in bacterial numbers	Aulik et al. (2010)
*Mycobacterium canettii*	Survives	Ramos-Kichik et al. (2009)
*Mycobacterium tuberculosis*	Survives	Ramos-Kichik et al. (2009)
*Pasteurella multocida*	Reduction in bacterial numbers	Scapinello et al. (2011)
*Porphyromonas gingivalis*	Survives	Delbosc et al. (2011); Palmer et al. (2011)
*Pseudomonas aeruginosa*	Survives	von Köckritz-Blickwede et al. (2008); Douda et al. (2011); Young et al. (2011); Khatua et al. (2012)
*Salmonella typhimurium*	Reduction in bacterial numbers	Brinkmann et al. (2004)
*Shigella flexneri*	Reduction in bacterial numbers	Brinkmann et al. (2004)
*Staphylococcus aureus*	Dependent on ratio	Döring et al. (2011)
*Staphylococcus epidermidis*	Survives	Cogen et al. (2010)
*Streptococcus pneumonia*	Survives	Beiter et al. (2006); Wartha et al. (2007); Midon et al. (2011)
*Streptococcus pyogenes*	Reduction in bacterial numbers	von Köckritz-Blickwede et al. (2008)
*Streptococcus suis*	Reduction in bacterial numbers	Scapinello et al. (2011)
*Yersinia enterocolitica*	Reduction in bacterial numbers	Casutt-Meyer et al. (2010)
*Yersinia pestis*	Survives	Casutt-Meyer et al. (2010)
**PROTOZOA**		
*Eimeria bovis*	Reduction in parasite numbers	Behrendt et al. (2010)
*Leishmania amazonesis*	Dependent on ratio	Guimarães-Costa et al. (2009)
*Leishmania donovani*	Survives	Gabriel et al. (2010)
*Plasmodium falciparum*	Trapped	Baker et al. (2008)
*Toxoplasma gondii*	Reduction in parasite numbers	Abi Abdallah et al. (2012)
**FUNGI**		
*Aspergillus fumigatus*	Growth inhibited	McCormick et al. (2010)
*Aspergillus nidulans*	Growth inhibited	Bianchi et al. (2011)
*Candida albicans*	Growth inhibited, blastospores survive	Urban et al. (2006); Menegazzi et al. (2012)
*Candida glabrata*	Growth inhibited	Springer et al. (2010)
*Cryptococcus gatti*	Survives	Springer et al. (2010)

## Concluding Perspective

Neutrophil extracellular traps have been suggested as an alternative or additional component of the innate host defense against microorganisms. Although progress has been made, many questions related to NET formation and function remain unanswered. Do NETs commonly occur *in vivo*? Compelling evidence is lacking. Are NETs formed by live neutrophils or does the process (i.e., NETosis) always result in cytolysis? If it is accompanied by neutrophil lysis, how does this phenomenon fit with what we know about the control of neutrophil turnover and the host efforts to prevent inflammatory syndromes? Importantly, is the pathway that leads to the formation of NETs a host-directed mechanism or simply an incidental phenomenon in neutrophils? These and other questions can only be answered by continued investigation into the biology and function of NETs.

## Conflict of Interest Statement

The authors declare that the research was conducted in the absence of any commercial or financial relationships that could be construed as a potential conflict of interest.
